# A bibliometric analysis of the relationship between traumatic brain injury and Alzheimer’s disease (1993-2023)

**DOI:** 10.3389/fnagi.2024.1462132

**Published:** 2024-10-23

**Authors:** Ji-Hua Hu, Xin Zhang, Hong-Mei Yang, Ya-Ling Xu, Ming Zhang, Xuan Niu

**Affiliations:** ^1^Department of Medical Imaging, The First Affiliated Hospital of Xi’an Jiaotong University, Xi’an, China; ^2^School of Future Technology, Xi’an Jiaotong University, Xi’an, China; ^3^Department of Pharmacy, Xi’an Honghui Hospital, Xi’an, China; ^4^Centre for Clinical Skills Training, Xi’an Children’s Hospital, Xi’an, China; ^5^Department of Rehabilitation Medicine, The First Affiliated Hospital of Xi’an Jiaotong University, Xi’an, China

**Keywords:** Alzheimer’s disease, traumatic brain injury, bibliometric analysis, Bibliometrix R, VOSviewer

## Abstract

**Background:**

Traumatic brain injury (TBI) increases the risk of developing Alzheimer’s disease (AD), and a growing number of studies support a potential link between the two disorders. Therefore, the objective of this study is to systematically map the knowledge structure surrounding this topic over the past and to summarize the current state of research and hot frontiers in the field.

**Methods:**

Data were retrieved from the Web of Science Core Collection (WOSCC) starting from the beginning until December 31, 2023, focusing on articles and reviews in English. Bibliometric tools including Bibliometrix R, VOSviewer, and Microsoft Excel were utilized for data analysis. The analysis included citations, authors, institutions, countries, journals, author keywords, and references.

**Results:**

A total of 1,515 publications were identified, comprising 872 articles (57.56%) and 643 reviews (42.44%). The annual number of publications increased steadily, especially after 2013, with an R^2^ value of 0.978 indicating a strong upward trend. The USA was the leading country in terms of publications (734 articles), followed by China (162 articles) and the United Kingdom (77 articles). Meanwhile Boston University was the most productive institution. Collaborative networks show strong collaborative author links between the USA and the United Kingdom, as well as China. The analysis also showed that the *Journal of Alzheimer’s Disease* was the most productive journal, while the article authored by McKee achieved the highest local citations value. The top three author keywords, in terms of occurrences, were “Alzheimer’s disease,” “traumatic brain injury,” and “neurodegeneration.” Thematic mapping showed a consolidation of research themes over time, decreasing from 11 main themes to 8. Emerging themes such as “obesity” and “diffusion tensor imaging” indicate new directions in the field.

**Conclusion:**

The research on AD after TBI has attracted a great deal of interest from scientists. Notably, the USA is at the forefront of research in this field. There is a need for further collaborative research between countries. Overall, this study provides a comprehensive overview of developments in TBI and AD research, highlighting key contributors, emerging topics, and potential areas for future investigation.

## 1 Introduction

Traumatic brain injury (TBI) has the highest incidence of all common neurological disorders (Maas et al., 2022), posing a global public health concern due to its association with prolonged disability, cognitive decline, and substantial healthcare costs (Guan et al., 2023; Tachino et al., 2024). Beyond the effects of acute injury, even a single acute TBI event can trigger long-term neurodegeneration processes, including Alzheimer’s disease (AD), Parkinson’s disease and chronic traumatic encephalopathy (Mao et al., 2020; Brody, 2011). Numerous studies have reported an increased prevalence of AD among individuals with a history of TBI, suggesting that TBI may serve as an important epigenetic risk factor for the development of AD and dementia later in life (Leung et al., 2022). Unfortunately, strategies that prevent or treat the clinical progression of AD have remained elusive (Self and Holtzman, 2023), significantly impacting patients’ quality of life and placing a substantial burden on healthcare systems. Thus, given the high prevalence of TBI, with an estimated 69 million cases annually (Dewan et al., 2019), and its associated neurodegenerative risk, investigating the link between TBI and AD is of critical medical and societal importance.

Emerging evidence have suggested a significant overlap in the pathophysiological processes of TBI and AD, supporting the biological mechanisms linking TBI to the development of AD. The pathogenesis of AD in the context of TBI involves the cleavage of amyloid precursor protein and tau protein through the activation of delta-secretase, thereby contributing to AD pathology (Wu et al., 2020). Consistent with the findings that tau is the primary structural component of neurofibrillary tangles (NFTs) in AD, TBI has been shown to induce the abnormal axonal accumulation of multiple kinases responsible for tau phosphorylation (Brett et al., 2022). Despite substantial research confirming TBI as a risk factor for AD, the findings remained inconsistent possibly due to the heterogeneity in study designs (Graham and Sharp, 2019), populations (Hicks et al., 2023), and methodologies (Hicks et al., 2019). More specifically, cognitive recovery after TBI can be influenced by a complex interaction of confounding factors, such as injury severity, time since injury, age, gender, genetic predisposition, lifestyle differences, and coping style (Raza et al., 2021; Ponsford, 2013).

To bridge existing gaps and assist researchers in the development of novel research topics, there is a critical need for more comprehensive and integrative research to clarify the relationship between TBI and AD. Beyond the synthesis of findings presented in reviews and meta-analyses, bibliometric analysis has a unique advantage of providing a comprehensive overview of the field of study. This approach synthesizes a large number of scientific publications and provides better insights into the knowledge structure and emerging research trends in specific research areas (Donthu et al., 2021). In recent years, this research approach has been previously employed to elucidate the progression of research in the domains of synaptic plasticity and AD (Zhang et al., 2023), neuroinflammation and AD (Sun et al., 2024), as well as the role of glial fibrillary acidic protein in AD (Zou et al., 2023).

Overall, given the growing body of research on TBI and its association with AD, a comprehensive bibliometric analysis can provide valuable insights into the current state of knowledge, identify research trends, and highlight gaps that need to be addressed. Despite rapid advancements in AD research, there remains a need for a thorough exploration of how the timing and severity of TBI influence AD development. Therefore, the objective of this study is to systematically map the knowledge structure surrounding this specific aspect of the TBI-AD relationship over the past and to forecast emerging research trends.

## 2 Materials and methods

### 2.1 Data sources

The data for this study was obtained from the WOSCC. In order to guarantee the precision and accuracy of retrieval, the Citation Index was configured to SCI-Expanded (SCI-E).

### 2.2 Search strategy

**FIGURE 1:**
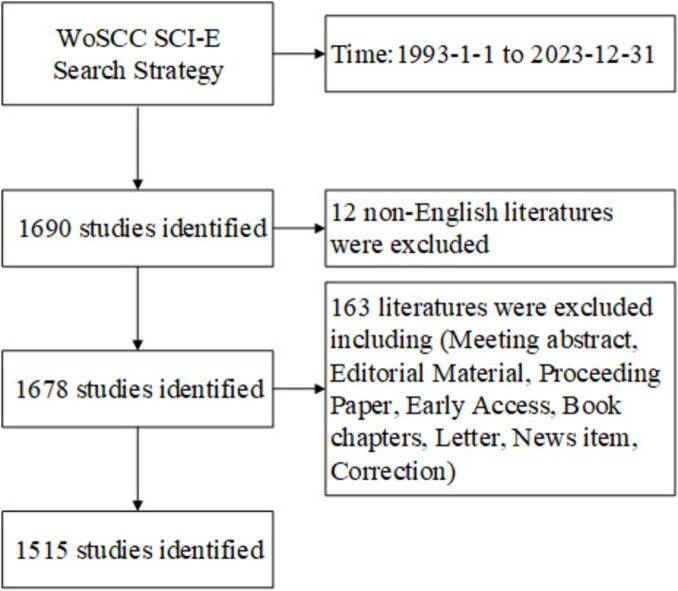
Data collection process.

For accuracy assurance, the search terms were utilized in advanced search with the title (TI), abstract (AB), and author keywords (AK) in advanced search (Ho et al., 2021; Fu et al., 2012). The search string for this study was set as follows: TI = (“traumatic brain injury”) OR AB = (“traumatic brain injury”) OR AK = (“traumatic brain injury”) and TI = (“Alzheimer’s disease”) OR AB = (“Alzheimer’s disease”) OR AK = (“Alzheimer’s disease”). Detailed retrieval of TBI and AD is included in Supplementary material. As seen in Figure 1, the search was conducted from January 1, 1993, to December 31, 2023. Refining the results involved filtering out other types of documents and only including English-language articles or reviews. 1,515 publications associated with TBI and AD were identified in the WOSCC SCI-E from 1993 to 2023, of which 872 (57.558%) were indexed as “article” and 643 (42.442%) as “review.” The results were exported in text format as “Full Record and Cited References.” Data were retrieved from WOSCC SCI-E on March 17, 2024.

### 2.3 Data analysis

The data analysis was conducted using Bibliometrix R (version 4.3.3) (Aria and Cuccurullo, 2017), VOSviewer (version 1.6.20) (van Eck and Waltman, 2010), and Microsoft Excel 2021 (Microsoft Corp., Redmond, WA, USA). The study utilized Bibliometrix R to gather descriptive bibliometric data and analyze the development of AD and TBI through conceptual themes and trends, including visualization of reference co-citation, keyword co-occurrence, co-authorship of countries/regions, etc. Among them, co-citation analysis was applied to examine the relationships between frequently cited papers, while co-authorship analysis was performed to map collaborations among countries/regions. Keyword co-occurrence analysis was also conducted to identify popular research topics and their evolution over time. Keywords appearing at least five times per year during the study period (1993–2023) were included in the analysis to capture the most influential themes. Additionally, VOSviewer was used to extract information such as the average normalized citations and average publication year (APY) for the included works. Microsoft Excel 2021 was utilized to chart the yearly scientific output and overall citation counts. To enhance the clarity of research presentation, the consolidation of synonyms is conducted prior to each visualization, ensuring uniformity in data depiction. This step is essential for the precise representation of data, achieved by unifying singular and plural forms, standardizing full names alongside their abbreviations, and normalizing letter case to either lowercase or uppercase.

## 3 Results

### 3.1 Analysis of annual publication distribution

**FIGURE 2:**
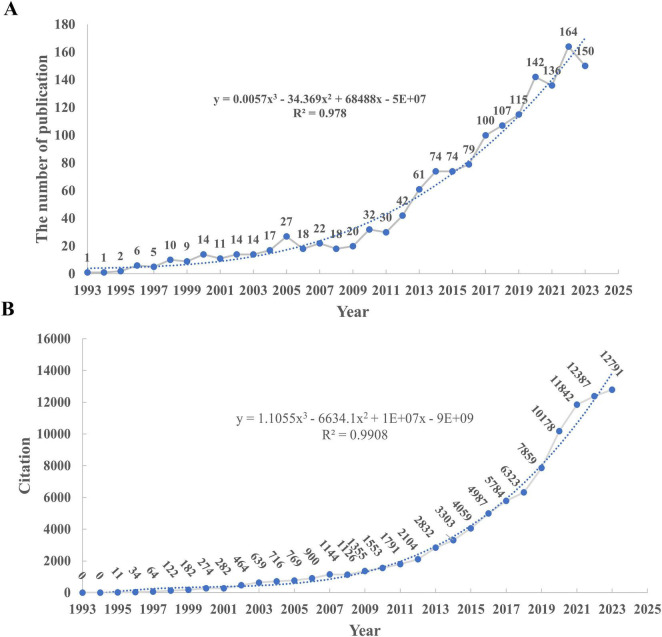
**(A)** Annual scientific production in the domains of TBI and AD. **(B)** Annual total citation in the domains of TBI and AD.

Figure 2A shows the number and trend of the annual publications obtained from the WOSCC. The first article was published in 1993, and from that year until 2013, there were 61 publications, a steady increase in the number of publications. The total number of publications increased steadily from 2013 to 2023. During this period, scholars have been paying increasing attention to TBI and AD. Furthermore, the trend was modeled using a curve fit, which achieved an R^2^ value of 0.978. The prediction curve indicates that there will be 204 publications on TBI and AD by 2025, continuing the trend of increasing literature in these areas.

The number of citations from the WOSCC’s citation report is displayed in Figure 2B, and it increases annually. There have been 97,815 citations in total, with an h-index of 151 and an average of 64.56 citations per article. The citation trend was modeled with a curve fit, resulting in an R^2^ value of 0.9908, indicating an exponentially increasing trend that aligns closely with the growth in published articles. This exponential trend has been particularly noticeable since 2013.

### 3.2 Descriptive bibliometric analysis results

#### 3.2.1 Summary bibliographic information

Table 1 displays the selected dataset, which consists of 1,509 articles published in 520 journals with an average publication date of 7.68 years. There are 64.82 citations on average per article. In the current dataset, there are 3,357 and 4,735 total author keywords and keywords plus, respectively. There are 7,435 contributors in all; 88 of them contributed as single authors and 7,347 as co-authors to the works.

**TABLE 1:** Main bibliographic information about the final dataset of the study.

Description	Results
Timespan	1993:2023
Number of journals	520
Number of articles	1509
Annual growth rate %	18.65
Average citations per article	64.82
Average years from publication	7.68
Number of references	113927
Number of keywords plus	4735
Number of author’s keywords	3357
Number of authors	7435
Authors of single-authored articles	88
Authors of multi-authored articles	7347
Number of single-authored articles	97
Co-authors per article	6.34
International co-authorships %	24.52

#### 3.2.2 Core journals

1,509 publications about TBI and AD were published in 520 journals between 1993 and 2023. The top 20 most prolific journals are included in Table 2, along with their total citations, APY, average normalized citations, and h-index. Average normalized citations solve the issue where more recent works haven’t had as much time to accumulate citations as previous ones (van Eck and Waltman, 2010). A journal’s APY is determined by summing up all of its publications within the topic under study. When a journal has h papers that each have at least h citations in the topic being studied, the journal’s h-index is h (Bihari et al., 2021).

**TABLE 2:** Top 20 most productive publication sources within the study areas of TBI and AD.

No.	Journal	Articles	Total citations	Average normalized citations	Average publication year	h-index
1	Journal of Alzheimer’s Disease	59	1398	0.43	2017.68	23
2	Journal of Neurotrauma	51	1842	0.46	2014.61	24
3	International Journal of Molecular Sciences	34	850	1.12	2021.09	15
4	Frontiers in Neurology	29	1019	0.61	2019.14	16
5	Frontiers in Neuroscience	28	671	0.64	2020.21	15
6	PLoS One	23	782	0.44	2016.39	15
7	Alzheimer’s & Dementia	22	1281	0.96	2017.00	18
8	Molecular Neurobiology	22	995	1.29	2018.05	14
9	Experimental Neurology	19	2501	1.38	2011.16	17
10	Frontiers in Aging Neuroscience	19	1050	1.01	2018.53	13
11	Brain	18	5127	3.13	2016.11	16
12	Acta Neuropathologica	17	2006	1.97	2015.18	16
13	Journal of Neuroscience	16	2450	1.63	2009.75	14
14	Journal of Neuroinflammation	16	1131	1.33	2016.81	13
15	Journal of Neuroscience Research	14	861	0.84	2011.64	10
16	Neurobiology of Disease	14	366	0.69	2018.43	10
17	Neural Regeneration Research	14	411	2.03	2020.71	9
18	Neuroscience	13	1004	0.77	2009.92	12
19	Acta Neuropathologica Communications	13	291	0.82	2021.15	8
20	Alzheimer’s Research & Therapy	13	597	0.68	2018.77	7

The *Journal of Alzheimer’s Disease* published the highest number of articles, accounting for around 3.91% of all publications. Followed by *Journal of Neurotrauma*, with 51 published articles. Compared to other journals, *Brain* has much more citations overall. The *Journal of Neurotrauma* has a considerably higher h-index than the other journals. The APY data for the journals listed indicates that *Journal of Neuroscience* published the most of its articles on TBI and AD much earlier (APY: 2009.75) compared to other journals, while *Acta Neuropathologica Communications* has published the majority of its articles in these areas more recently (APY: 2021.15).

**FIGURE 3:**
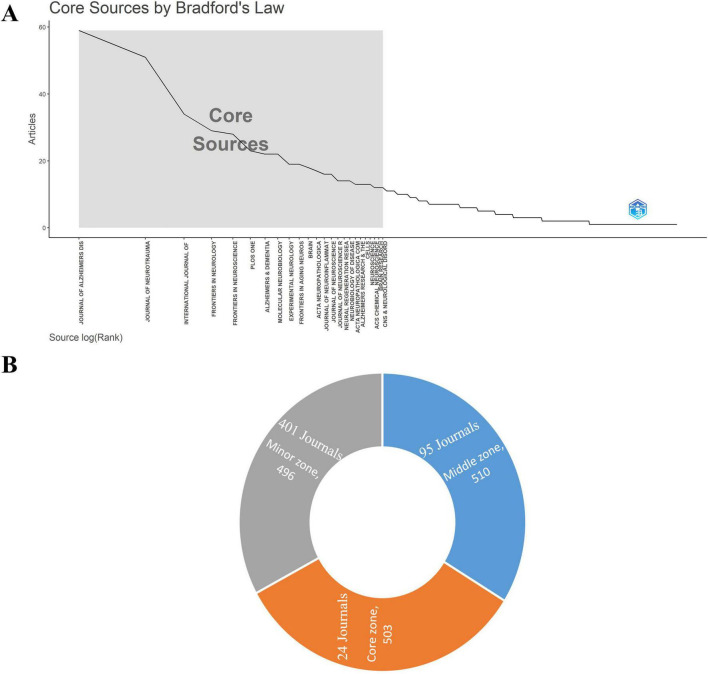
**(A,B)** Core journals contributing to the publication of articles in the study areas of TBI and AD based on Bradford’s law.

According to Bradford’s law, Figures 3A, B the core zone consists of 24 high quality journals with 503 articles published, notably the *Journal of Alzheimer’s Disease*, *Journal of Neurotrauma*, and *International Journal of Molecular Sciences*. The middle and minor zones contain 510 and 496 papers, respectively, from 95 to 401 different journals.

#### 3.2.3 Geographic distribution of published articles

**FIGURE 4:**
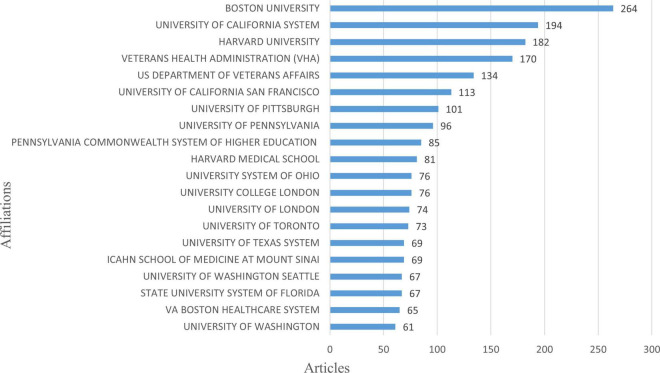
The most contributing institutions to the literature on TBI and AD.

The affiliations of all authors involved in the article were considered to evaluate the collaboration and involvement of countries and organizations in this part. The top contributing institutions to the study of AD and TBI between 1993 and 2023 are displayed in Figure 4. Boston University is the institution that has produced the most with 264 publications. This institution is followed by the University of California System with 194 papers and Harvard University with 182 papers, respectively.

**FIGURE 5:**
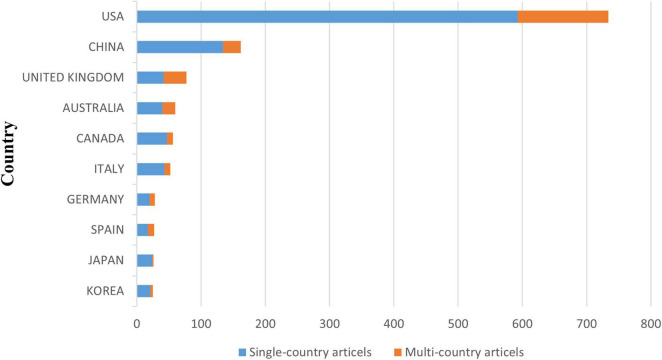
Geographical distribution of the published articles based on the corresponding author’s country.

Based on the affiliations of the corresponding authors as stated in the published articles, Figure 5 lists the top 10 contributing countries. In terms of publications, the USA topped the list with 734, followed by China with 162 and the United Kingdom with 77. The USA recorded the highest production of single-country (593 articles) and multiple-country (141 articles) publications. The United Kingdom had the highest ratio of publications involving multiple countries.

**FIGURE 6:**
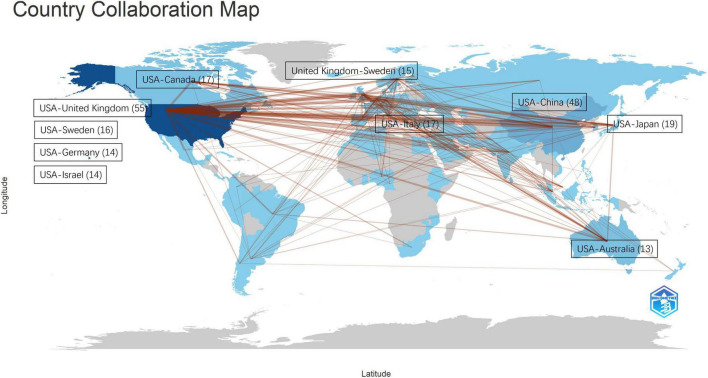
The most frequent co-authorship collaborations among the contributing countries in the fields of TBI and AD.

With a frequency of at least five, Figure 6 illustrates the co-authorship relationships among the contributing nations to the literature under study and indicates the most frequent collaboration links. The most frequent collaboration connection, rated at 55, exists between the USA and the United Kingdom. Of the contributing countries, the collaboration between the USA and China has the second strongest co-authorship link with a frequency of 48.

As shown in Table 3, the USA and China rank first and second, respectively, out of 57 countries, with 832 and 182 published articles, respectively. The top influential countries are Norway, the Netherlands, and Denmark, with average normalized citations of 3.05, 2.40, and 1.92, in that order.

**TABLE 3:** The list of top 10 countries in terms of scientific production and average normalized citations in the fields of TBI and AD.

Rank	Country	Articles	Rank	Country	Average normalized citations
1	USA	832	1	Norway	3.05
2	China	182	2	Netherlands	2.40
3	England	110	3	Denmark	1.92
4	Italy	77	4	Scotland	1.69
5	Australia	73	5	Austria	1.59
6	Canada	71	6	England	1.55
7	Germany	53	7	Wales	1.49
8	Japan	52	8	Switzerland	1.35
9	Sweden	47	9	Chile	1.28
10	Spain	42	10	Germany	1.22

#### 3.2.4 Most influential articles

Local citation (LC) and global citation (GC) scores were used in this study’s analysis of the collected articles. GC represents the total citations an article has received across all databases, while LC shows the citations an article has received from articles within the dataset being studied.

Tables 4–6 rank the top 10 articles in the dataset being studied based on GC, LC, and average normalized citations, as per the current research. Table 6 indicates that the top 10 most referenced articles, based on the average normalized citation score, were featured in 8 distinct publications. *Glutamate Uptake* (Danbolt, 2001) is the article with the highest GC value among the top 10 most cited articles worldwide. The article with the highest LC value was written by McKee et al. (2013). Ikonomovic MD authored the article with the highest LC/GC ratio. Research conducted by Feigin et al. is highly cited according to the average normalized citations.

**TABLE 4:** The top 10 most globally cited articles within the domains of TBI and AD.

Author	Title	Journal	GC	GC per year
Danbolt NC. 2001	Glutamate uptake	Progress in Neurobiology	3636	151.50
Feigin VL. 2019	Global, regional, and national burden of neurological disorders, 1990-2016: a systematic analysis for the Global Burden of Disease Study 2016	The Lancet. Neurology	2051	341.83
Pandharipande PP. 2013	Long-term cognitive impairment after critical illness	The New England Journal of Medicine	1635	136.25
Leech R, 2014	The role of the posterior cingulate cortex in cognition and disease	Brain	1423	129.36
McKee AC. 2013	The spectrum of disease in chronic traumatic encephalopathy	Brain	1391	115.92
Sweeney MD, 2019	Blood-Brain Barrier: From Physiology to Disease and Back	Physiological Reviews	1039	173.17
Lucas SM. 2006	The role of inflammation in CNS injury and disease	British Journal of Pharmacology	996	52.42
Amor S. 2010	Inflammation in neurodegenerative diseases	Immunology	963	64.20
Bell RD. 2012	Apolipoprotein E controls cerebrovascular integrity via cyclophilin A	Nature	911	70.08
Stam CJ. 2014	Modern network science of neurological disorders	Nature Review. Neuroscience	830	75.45

GC, global citation.

**TABLE 5:** The top 10 most locally cited articles in the studied dataset on TBI and AD.

Author	Title	Journal	LC	GC	LC/GC ratio (%)
McKee AC. 2013	The spectrum of disease in chronic traumatic encephalopathy	Brain	173	1391	12.44
Ikonomovic MD. 2004	Alzheimer’s pathology in human temporal cortex surgically excised after severe brain injury	Experimental Neurology	114	331	34.44
Johnson VE. 2012	Widespread τ and amyloid-β pathology many years after a single traumatic brain injury in humans	Brain Pathology	113	441	25.62
Johnson VE. 2010	Traumatic brain injury and amyloid-β pathology: a link to Alzheimer’s disease?	Nature Review. Neuroscience	98	421	23.28
McKee AC. 2016	The first NINDS/NIBIB consensus meeting to define neuropathological criteria for the diagnosis of chronic traumatic encephalopathy	Acta Neuropathologica	80	575	13.91
Uryu K. 2007	Multiple proteins implicated in neurodegenerative diseases accumulate in axons after brain trauma in humans	Experimental Neurology	74	288	25.69
Nemetz PN. 1999	Traumatic brain injury and time to onset of Alzheimer’s disease: a population-based study	American Journal of Epidemiology	72	230	31.30
Omalu BI. 2005	Chronic traumatic encephalopathy in a National Football League player	Neurosurgery	70	686	10.20
Tran HT. 2011	Controlled cortical impact traumatic brain injury in 3xTg-AD mice causes acute intra-axonal amyloid-β accumulation and independently accelerates the development of tau abnormalities	The Journal of Neuroscience	66	199	33.17
Geddes JF. 1999	Neuronal cytoskeletal changes are an early consequence of repetitive head injury	Acta Neuropathologica	61	242	25.21

GC, global citation; LC, local citation.

**TABLE 6:** The top 10 most influential articles within the fields of TBI and AD based on average normalized citations.

Reference	Title	Journal	Average normalized citations	GC
Feigin VL. 2019	Global, regional, and national burden of neurological disorders, 1990-2016: a systematic analysis for the Global Burden of Disease Study 2016	The Lancet. Neurology	27.5	2051
Shi Y. 2021	Structure-based classification of tauopathies	Nature	14.26	313
Sweeney MD. 2019	Blood-Brain Barrier: From Physiology to Disease and Back	Physiological Reviews	13.93	1039
Cornell J. 2022	Microglia regulation of synaptic plasticity and learning and memory	Neural Regeneration Research	12.53	131
Rasmussen MK. 2018	The glymphatic pathway in neurological disorders	The Lancet Neurology	12.17	696
Amanollahi M. 2023	The Dialogue Between Neuroinflammation and Adult Neurogenesis: Mechanisms Involved and Alterations in Neurological Diseases.	Molecular Neurobiology	9.78	29
Zhang WJ. 2020	Novel tau filament fold in corticobasal degeneration	Nature	9.17	310
McKee AC. 2013	The spectrum of disease in chronic traumatic encephalopathy	Brain	8.84	1391
Amor S. 2010	Inflammation in neurodegenerative diseases	Immunology	8.81	963
McKee AC. 2016	The first NINDS/NIBIB consensus meeting to define neuropathological criteria for the diagnosis of chronic traumatic encephalopathy	Acta Neuropathologica	8.54	575

GC, global citation.

### 3.3 Keywords frequency

**FIGURE 7:**
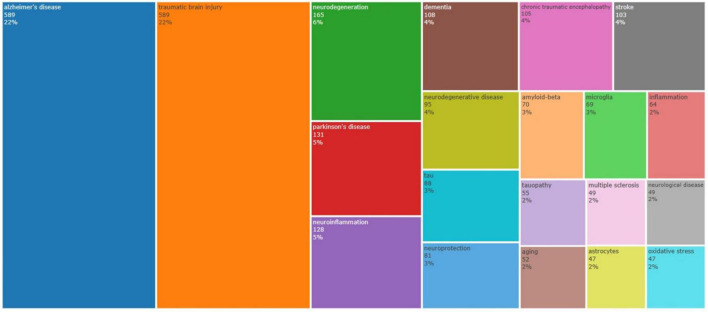
Tree map of the most frequent author keywords in the studied domain.

**FIGURE 8:**
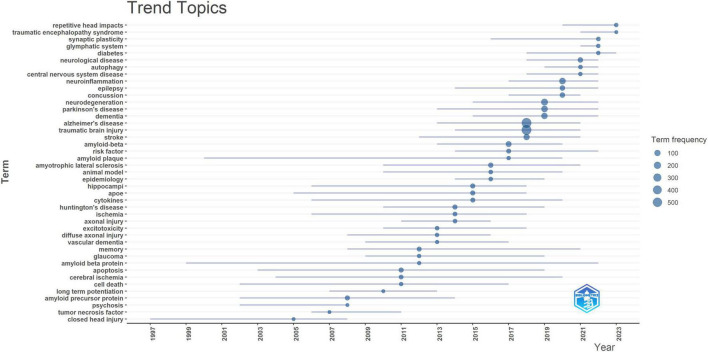
Trend topics in the research domains of TBI and AD.

The 20 most frequent author keywords used in the study areas of TBI and AD are presented in Figure 7. In the dataset under examination, the top three author keywords are “Alzheimer’s disease,” “traumatic brain injury,” and “neurodegeneration,” with 589, 589, and 165 instances, respectively, making up 22, 22, and 6% of all keyword occurrences. Moreover, “neuroinflammation” and “microglia” have 128 and 69 occurrences, respectively, with shares of 6 and 3%. Among the top 20 author keywords that were presented, “chronic traumatic encephalopathy,” “tau,” and “amyloid beta” were noted, with 105, 88, and 70 occurrences, respectively, these terms accounted for 4, 3, and 3% of the total terms that were presented. As shown in Figure 8, the main trend topics in 2023 were “repetitive head impacts” and “traumatic encephalopathy syndrome.” The trend topics in 2022 were “synaptic plasticity,” “glymphatic system,” and “diabetes.”

**FIGURE 9:**
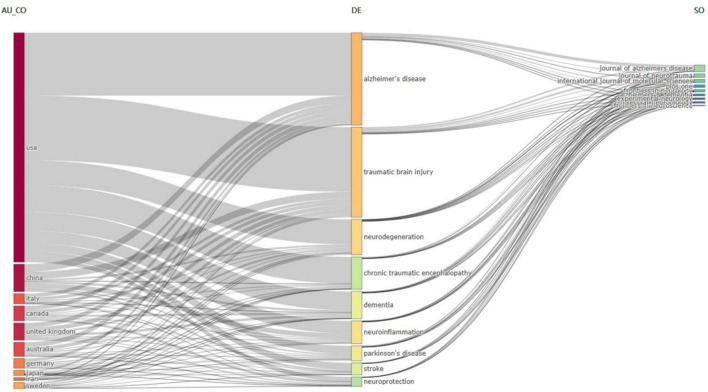
Three-fields plot linking author keywords with the relevant contributing countries and journals in the studied field.

The USA-affiliated researchers have focused their attention more on the most common author keywords compared to other countries, as illustrated in Figure 9. China and Italy are ranked second and third based on the sizes of their labels on the figure. The figure displays the connection between the author’s keywords and the journals that released the articles within the TBI and AD research field on the right side. The *Journal of Alzheimer’s Disease* stands out among other journals for having the largest proportion of published articles associated with author keywords, as shown by the size of the journal labels.

### 3.4 Thematic evolution in the fields of TBI and AD

**FIGURE 10:**
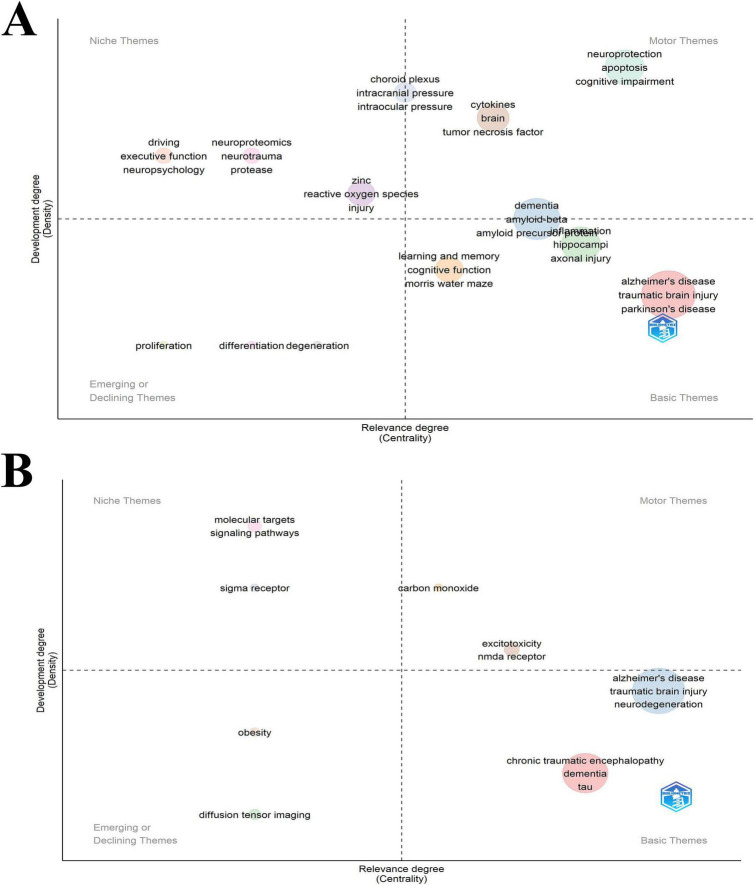
**(A)** Thematic evolution within the TBI and AD domains in the period 1993–2013. **(B)** Thematic evolution within the TBI and AD domains in the period 2014–2023.

Our data was analyzed using two time slices, which separated the sample under study into two segments covering the years 1993–2013 and 2014–2023. A thematic map was constructed for each time slice, as Figures 10A, B illustrate. Based on clusters of keywords, these thematic plots provide a strategic map. The degree of development increases with density, and the degree of relevance increases with centrality (Agbo et al., 2021).

From 1993 to 2013, the research on TBI and AD identified 11 main themes in its conceptual framework. But from 2014 to 2023, this number dropped to 8 with smaller clusters. The figure highlighted a prominent theme related to “dementia, amyloid beta, and amyloid precursor protein” positioned between the upper-right and lower-right quadrants, which significantly influenced the research landscape from 1993 to 2013. In the period 2014–2023, themes such as “Alzheimer’s disease, traumatic brain injury, and neurodegeneration,” and “chronic traumatic encephalopathy, dementia, and tau” seen in the lower-right quadrant are the basics and are very important for the field’s development. Themes located in the upper-left quadrant have established internal connections but are only making a minimal impact on the advancement of TBI and AD research. This discovery indicates that subjects located in the upper-left quadrant, including “molecular targets, signaling pathways, and sigma receptor,” should be further integrated into studies on TBI and AD. The themes in the lower-left quadrant, “obesity, diffusion tensor imaging” appear to be emerging, demonstrating that some of its elements are fundamental and required for the advancement of the TBI and AD fields. The topics located in the top-right section, “carbon monoxide, excitotoxicity, and NMDA receptor,” are extensively explored and essential for the progress of research on TBI and AD.

## 4 Discussion

The analysis of the annual distribution and impact of publications on TBI and AD highlights several significant trends and implications within the research community. The steady increase in publications since 1993, with an accelerated growth observed from 2013 onward, underscores the escalating scientific and clinical interest in these areas. This growth is likely influenced by the aging global population and the corresponding rise in neurodegenerative conditions, enhancements in funding allocations, heightened awareness of the long-term impacts of TBI, and advances in diagnostic technologies (Sahebnasagh et al., 2022; Dams-O’Connor et al., 2023). It is worth noting that the high R^2^ values observed for publication trends (0.978) and citation trends (0.9908) indicate a strong fit with the historical data, but also raise concerns about potential overfitting. The model might be overfitting past trends, potentially limiting its accuracy in forecasting future. Possible external influences, such as changes in citation practices, publication bias, and shifts in research funding, should be considered when interpreting the fitted model’s predictions.

The bibliometric analysis reveals significant insights into the publication trends for TBI and AD research across different journals from 1993 to 2023. The *Journal of Alzheimer’s Disease*, *Journal of Neurotrauma*, and *International Journal of Molecular Sciences* are highlighted as key publishers in the field. Of these, *Journal of Alzheimer’s Disease* leading in article volume, an international multidisciplinary journal to facilitate progress in understanding the etiology, pathogenesis, epidemiology, genetics, behavior, treatment and psychology of AD, become the major forum for publication in AD. Commendably, the editors of this the journal have a relatively even gender ratio and are widely distributed throughout the world. This indicates a concentrated effort within the journal to advance research in these areas. The use of metrics like average normalized citations and h-index helps to understand the impact and reach of the research published, showing that more recent publications can still be influential despite shorter citation windows. Meanwhile, a high h-index and average citation per article indicating substantial influence and recognition within the scientific community. The exponential increase in citations, particularly post-2013, could be reflective of seminal works that have significantly advanced the field, influencing subsequent studies and practices. Additionally, the APY data suggest shifts in research focus over time, with some journals publishing foundational research earlier and others contributing newer findings. This distribution of publications, as described by Bradford’s law (Chen et al., 2024), underscores the central role that a core group of journals plays in disseminating influential research, while also highlighting the broad, diverse contribution of many other journals in the field.

This study demonstrates the steady development of research on AD after TBI and the increasing number of international collaborations. With a total of 832 publications, the USA is the dominant country in this field. In addition, the top 10 institutions in the field are all located in the USA, further strengthening its influence in the field. Notably, this result is consistent with the bibliometric results of Sang et al.’s (2023) literature on post-TBI dementia, revealing that the USA has placed great emphasis on neurodegenerative disorders with more in-depth research. Moreover, the strong collaborative links between countries, especially between the USA and the UK, also underline the global nature of research efforts and the importance of international collaboration in tackling the complex challenges posed by TBI and AD.

In addition, the article with the highest LC value was written by McKee et al. (2013) (McKee et al., 2013). Combined with other literature citation analyses, it can be seen that McKee AC is a leading researcher in this field and has been focusing on AD research, directing the Brain Banks for the Boston University Alzheimer’s Disease Center. Her research interests center on the neuropathological alterations of neurodegenerative diseases, with a primary focus on the role of tau protein, axonal injury, trauma, vascular injury, and neurodegeneration. Much of her current work centers on mild traumatic brain injury from contact sports and military service and its long-term consequences. Next, co-authorship analysis showed that the USA was involved in almost all collaborations and was mostly in the lead. The prominent collaboration between the USA and the United Kingdom, with a frequency of 55, highlights a strong transatlantic partnership that may significantly influence the research landscape. Similarly, the substantial co-authorship link between the USA and China, rated at 48, indicates a growing synergy between these major contributors. Future studies could benefit from a longitudinal analysis of co-authorship patterns to assess the progression and impact of these collaborations, thus providing a comprehensive understanding of the role of international collaborations in advancing the field.

Keyword analysis shows that “Alzheimer’s disease” and “traumatic brain injury” each account for 22% of the total keyword occurrences, indicating that research in this field is highly concentrated on these core topics. In contrast, in other medical research areas like cancer research or infectious diseases, keywords may be more diverse, covering a broader range of subtopics. This high concentration of keywords may reflect the specialization of the TBI and AD research field but may also suggest limitations in research perspectives. Additionally, “neurodegeneration” ranks third, emphasizing the focus on the neuropathological mechanisms of these diseases. Furthermore, the high frequency of “neuroinflammation” and “microglia” reflects researchers’ emphasis on the role of inflammatory responses in TBI and AD. The high occurrence of these keywords indicates that exploring the pathophysiological processes of the diseases is an important research direction in this field, aiding in the development of new therapeutic strategies. However, compared to fields with close interdisciplinary collaboration, such as bioinformatics or systems biology, TBI and AD research seems more inclined to delve deeply into specific biological mechanisms, lacking integration with other disciplines. This may lead to fragmentation within the research community, limiting the introduction of new methods and multidisciplinary perspectives.

It is worth noting that a strong correlation was found between chronic traumatic encephalopathy (CTE) and repeated head injuries (McKee et al., 2023). Only a postmortem neuropathological examination can provide a definitive diagnosis of CTE; the corresponding clinical condition was called traumatic encephalopathy syndrome (Katz et al., 2021; Cullum and LoBue, 2021; Hazrati and Schwab, 2021). The loss of excitatory synapses is known to underlie the cognitive deficits in AD (Bhembre et al., 2023; Cornell et al., 2022). Novel synaptic indicators are currently in development to assist in the early detection of AD (Tzioras et al., 2023; Colussi et al., 2023; Dai et al., 2024). Amyloid pathology may be preceded by lymphatic failure, which also serves as a predictor of amyloid deposition, neurodegeneration, and clinical progression in AD (Huang et al., 2024). Research has indicated that the progression of AD is influenced by chronic inflammation and compromised insulin signaling (Shen et al., 2022). Additionally, in the thematic analysis, we observed that the number of main themes decreased from 11 to 8. This reduction may reflect a consolidation of knowledge, where researchers have reached consensus on key areas as research deepens, leading to a more focused research emphasis. It could also indicate a shift in research priorities; with new discoveries and technologies, researchers are concentrating more on specific hotspot areas. Additionally, changes in funding landscapes might have influenced the intensity of research on certain topics, leading to a decrease in the number of themes (Rafols et al., 2010).

Regarding emerging themes, the appearance of “obesity” and “diffusion tensor imaging” is noteworthy. The emergence of “obesity” as a theme may reflect increasing interest in the relationship between metabolic disorders and neurodegenerative diseases, suggesting that obesity might be a risk factor or influencing factor in the development of TBI and AD (Anstey et al., 2011; Whitmer et al., 2005). “Diffusion tensor imaging” (DTI), as an advanced neuroimaging technology, is gaining importance, indicating a demand for more precise diagnostic and research tools. DTI helps reveal changes in the brain’s white matter structure, providing detailed information about microstructural brain damage in patients with TBI and AD. This could potentially promote the development of early diagnosis and personalized treatment plans (Hulkower et al., 2013; Nir et al., 2013). Overall, In-depth exploration of “obesity” could promote research into preventive strategies, while the application of “diffusion tensor imaging” may accelerate the understanding of disease mechanisms. Therefore, focusing on and developing these areas can enrich perspectives in TBI and AD research, promote multidisciplinary collaboration, and drive innovation and progress in the field.

## 5 Limitations

There are some limitations to the systematic science mapping conducted in this research. The constraints primarily stem from the database and techniques employed in science mapping analysis. First, we used only the WOSCC database as a data source, excluding other medical databases such as PubMed or Scopus. However, the quantity and quality of the WOSCC database search results are generally recognized, and the amount of data we analyzed would be adequate to reflect the true state of the TBI and AD research field. Additionally, only English-language publications were included, introducing the potential for language bias, as relevant research published in other languages may have been overlooked. In addition, Bibliometrix R and VOSviewer were used for bibliometric analysis in this study. In the future, conduct comparable bibliometric analyses could be conducted using other software packages, such as CiteSpace, to improve the robustness of the results. The exclusion of gray literature is another limitation, as it may have resulted in the omission of potentially valuable insights from non-peer-reviewed sources. These factors should be considered when interpreting the findings, and future work could address these limitations by broadening the database and software scope.

## 6 Conclusion

The occurrence of AD after TBI has attracted research interest from neurologists worldwide, and research achievements in this field have been increasing per year. The USA is the leading country in almost every aspect of this field, including the number of publications, authors, and institutions. In the future, researchers in this field should strengthen international scientific cooperation, especially with leading countries like the USA. Furthermore, the hotspots in this field include “neurodegeneration,” “neuroinflammation,” “microglia,” “obesity,” and “diffusion tensor imaging.” Research on the pathological mechanisms in this field is also receiving increasing attention. In summary, this bibliometric analysis provides valuable insights into the current status and research hotspots of this field, which is expected to serve as a reference for researchers and policy makers.

## Data Availability

The original contributions presented in this study are included in the article/Supplementary material, further inquiries can be directed to the corresponding authors.
