# Supplementary material for: A bibliometric analysis of the relationship between traumatic brain injury and Alzheimer’s disease (1993-2023)
Source: Front Aging Neurosci. 2024 Oct 23;16:1462132. doi: 10.3389/fnagi.2024.1462132 (PMC11538086; doi:10.3389/fnagi.2024.1462132)
Supplement: Supplementary file 1 [file Table_1.DOCX]

**The search string for this study:**

((TI=(“Brain Injury, Traumatic” OR “Traumatic Brain Injur*” OR “Trauma*, Brain” OR “Brain Trauma*” OR “TBI” OR “Encephalopath*, Traumatic” OR “Traumatic Encephalopath*” OR “Injury, Brain, Traumatic”)) OR AB=(“Brain Injury, Traumatic” OR “Traumatic Brain Injur*” OR “Trauma*, Brain” OR “Brain Trauma*” OR “TBI” OR “Encephalopath*, Traumatic” OR “Traumatic Encephalopath*” OR “Injury, Brain, Traumatic”)) OR AK=(“Brain Injury, Traumatic” OR “Traumatic Brain Injur*” OR “Trauma*, Brain” OR “Brain Trauma*” OR “TBI” OR “Encephalopath*, Traumatic” OR “Traumatic Encephalopath*” OR “Injury, Brain, Traumatic”) and ((TI=(“Alzheimer Dementia*” OR “Dementia, Alzheimer” OR “Alzheimer's Disease” OR “Dementia, Senile” OR “Senile Dementia” OR “Dementia, Alzheimer Type” OR “Alzheimer Type Dementia” OR “Alzheimer-Type Dementia” OR “Dementia, Alzheimer-Type” OR “Alzheimer Type Senile Dementia” OR “Primary Senile Degenerative Dementia” OR “Dementia, Primary Senile Degenerative” OR “Alzheimer Sclerosis” OR “Sclerosis, Alzheimer” OR “Alzheimer Syndrome” OR “Alzheimer's Diseases” OR “Alzheimer Diseases” OR “Alzheimers Diseases” OR “Senile Dementia, Alzheimer Type” OR “Acute Confusional Senile Dementia” OR “Senile Dementia, Acute Confusional” OR “ Dementia, Presenile” OR “Presenile Dementia” OR “Alzheimer Disease, Late Onset” OR “Late Onset Alzheimer Disease” OR “Alzheimer's Disease, Focal Onset” OR “Focal Onset Alzheimer's Disease” OR “Familial Alzheimer Disease” OR “Alzheimer Disease, Familial” OR “Familial Alzheimer Diseases” OR “Alzheimer Disease, Early Onset” OR “Early Onset Alzheimer Disease” OR “Presenile Alzheimer Dementia”)) OR AB=(“Alzheimer Dementia*” OR “Dementia, Alzheimer” OR “Alzheimer's Disease” OR “Dementia, Senile” OR “Senile Dementia” OR “Dementia, Alzheimer Type” OR “Alzheimer Type Dementia” OR “Alzheimer-Type Dementia” OR “Dementia, Alzheimer-Type” OR “Alzheimer Type Senile Dementia” OR “Primary Senile Degenerative Dementia” OR “Dementia, Primary Senile Degenerative” OR “Alzheimer Sclerosis” OR “Sclerosis, Alzheimer” OR “Alzheimer Syndrome” OR “Alzheimer's Diseases” OR “Alzheimer Diseases” OR “Alzheimers Diseases” OR “Senile Dementia, Alzheimer Type” OR “Acute Confusional Senile Dementia” OR “Senile Dementia, Acute Confusional” OR “ Dementia, Presenile” OR “Presenile Dementia” OR “Alzheimer Disease, Late Onset” OR “Late Onset Alzheimer Disease” OR “Alzheimer's Disease, Focal Onset” OR “Focal Onset Alzheimer's Disease” OR “Familial Alzheimer Disease” OR “Alzheimer Disease, Familial” OR “Familial Alzheimer Diseases” OR “Alzheimer Disease, Early Onset” OR “Early Onset Alzheimer Disease” OR “Presenile Alzheimer Dementia”)) OR AK=(“Alzheimer Dementia*” OR “Dementia, Alzheimer” OR “Alzheimer's Disease” OR “Dementia, Senile” OR “Senile Dementia” OR “Dementia, Alzheimer Type” OR “Alzheimer Type Dementia” OR “Alzheimer-Type Dementia” OR “Dementia, Alzheimer-Type” OR “Alzheimer Type Senile Dementia” OR “Primary Senile Degenerative Dementia” OR “Dementia, Primary Senile Degenerative” OR “Alzheimer Sclerosis” OR “Sclerosis, Alzheimer” OR “Alzheimer Syndrome” OR “Alzheimer's Diseases” OR “Alzheimer Diseases” OR “Alzheimers Diseases” OR “Senile Dementia, Alzheimer Type” OR “Acute Confusional Senile Dementia” OR “Senile Dementia, Acute Confusional” OR “ Dementia, Presenile” OR “Presenile Dementia” OR “Alzheimer Disease, Late Onset” OR “Late Onset Alzheimer Disease” OR “Alzheimer's Disease, Focal Onset” OR “Focal Onset Alzheimer's Disease” OR “Familial Alzheimer Disease” OR “Alzheimer Disease, Familial” OR “Familial Alzheimer Diseases” OR “Alzheimer Disease, Early Onset” OR “Early Onset Alzheimer Disease” OR “Presenile Alzheimer Dementia”)
