# Supplementary material for: A bibliometric analysis of the relationship between traumatic brain injury and Alzheimer’s disease (1993-2023)
Source: Front Aging Neurosci. 2024 Oct 23;16:1462132. doi: 10.3389/fnagi.2024.1462132 (PMC11538086; doi:10.3389/fnagi.2024.1462132)
Supplement: Supplementary file 2 [file Table_2.DOCX]

Supplemental table for Fig.5. Geographical distribution of the published articles based on the corresponding author’s country.

| **Rank** | **Country** | **Articles** | **SCP** | **MCP** | **MCP_Ratio** |
| --- | --- | --- | --- | --- | --- |
| 1 | USA | 734 | 593 | 141 | 0.192 |
| 2 | China | 162 | 134 | 28 | 0.173 |
| 3 | United Kingdom | 77 | 42 | 35 | 0.455 |
| 4 | Australia | 60 | 40 | 20 | 0.333 |
| 5 | Canada | 56 | 47 | 9 | 0.161 |
| 6 | Italy | 52 | 43 | 9 | 0.173 |
| 7 | Germany | 28 | 20 | 8 | 0.286 |
| 8 | Spain | 27 | 17 | 10 | 0.37 |
| 9 | Japan | 26 | 24 | 2 | 0.077 |
| 10 | Korea | 25 | 21 | 4 | 0.16 |
